# Social vocalizations indicate behavioural type in *Glossophagine* bats

**DOI:** 10.1098/rspb.2024.2217

**Published:** 2025-01-29

**Authors:** Theresa Schabacker, Raffaella Castiglione, Lysanne Snijders, Mirjam Knörnschild

**Affiliations:** ^1^ Museum für Naturkunde, Leibniz-Institute for Evolution and Biodiversity Science, Invalidenstr 43, Berlin 10115, Germany; ^2^ Department of Biology, Chemistry and Pharmacy, Institute of Biology, Freie Universität, Altensteinstr 6, Berlin 14195, Germany; ^3^ Behavioural Ecology Group, Wageningen University and Research, De Elst 1, Wageningen 6708 WD, The Netherlands; ^4^ Faculty of Life Sciences, Department of Biology, Humboldt Universitӓt zu Berlin, Invalidenstr 110, Berlin 10115, Germany; ^5^ Smithsonian Tropical Research Institute, Luis Clement Avenue, Bldg. 401 Tupper, Balboa Ancon, Panama

**Keywords:** animal personality, communication, *Glossophaga* bats, consistent between-individual differences, vocalizations, behavioural syndrome

## Abstract

Vocalizations play a crucial role in the social systems of many animals and may inadvertently reveal behavioural characteristics of the sender. Bats, the second largest mammalian order, rely extensively on vocalizations owing to their nocturnal lifestyle and complex social systems, making them ideal for studying links between vocalizations and consistent behavioural traits. In this study, we developed a new testing regime to investigate whether consistent individual vocalization differences in nectarivorous bats are associated with specific behavioural types. We exposed 60 wild, male *Glossophaga soricina handleyi* bats to novel and risky stressors and assessed their behavioural and vocal responses. Proactive, exploratory and bold bats were more likely to produce social calls, and among the vocalizing bats, more agitated bats produced higher numbers of social calls. We thus show that bat vocalization behaviour can be indicative of a certain behavioural type, potentially allowing conspecifics to assess personalities from a distance, which in turn could impact subsequent social interactions, group dynamics and reproductive success. Our results, in combination with previous findings in birds, suggest that advertent or inadvertent long-distance broadcasting of personality may be widespread, thus opening up new exciting questions about the links between vocalizations and sociality.

## 1. Introduction

Across taxa, vocalizations serve fundamental roles in the lives of animals [1,2], functioning in mediating mating opportunities [3], conveying information about individual qualities [4] or emotional states (reviewed in [5,6]), facilitating collective movement and group cohesion [2,7], strengthening social bonds [8] or aiding in orientation and navigation [9]. Despite its pervasive relevance, vocalizations have been notably understudied in the context of consistent between-individual differences, i.e. animal personality traits, and existing research is heavily biased towards birds. For example, in male great tits (*Parus major*), faster explorers have higher song rates before the egg-laying period [10] and generally respond more strongly vocally [11], and males with higher singing activity have higher reproductive success [12]. In black-capped chickadees (*Poecile atricapillus*), more exploratory birds produce more calls in stressful situations [13]. Beyond birds, vocalizations aid in assessing emotional reactivity and arousal states in farm animals and other mammals [5,6].

Bats, the second largest order of mammals, are highly vocal animals, in part owing to their nocturnality and unique life histories. Bats display a remarkable variability in roosting and feeding resources. Many bat species rely on insect swarms or fruiting and flowering trees, food sources that are often highly ephemeral and occur unpredictably but highly clumped, encouraging communication and eavesdropping among conspecifics [14,15]. Additionally, bats are highly social and many species live in colonies of several thousand individuals, often forming cohesive, long-term social bonds with members of their social groups [16]. All these life history traits point to the pivotal relevance of vocalizations as a key trait in a bat’s life. Bats employ two different types of vocalizations: on the one hand, echolocation calls serve the (primary) purpose of navigation and orientation in the environment and enable bats to detect, identify and capture prey in the dark with an extraordinarily high precision rate [17,18]. On the other hand, bats actively emit social vocalizations, which can occur either as calls (short, comparatively simple vocalizations) or songs (complex, multisyllabic, sexually selected vocalizations) with the sole purpose of communicating information to con- and heterospecifics [19]. In both echolocation and social calls, there is extensive inter- and intra-specific variation in acoustic features, suggesting that bats can discriminate easily between con- and heterospecifics, up to the individual level [20,21]. While much research effort has focused on elucidating this between-individual variation of acoustic features, almost nothing is known about between-individual differences in the production rates of either vocalization. Only a few exceptions exist: in a wild population of a temperate bat species, *Pipistrellus nathusii*, individuals consistently differ in ‘acoustic exploration’, a trait describing the number of echolocation calls emitted in a novel environment (NE), indicating that individual bats vary in their degree of environmental cue sampling [22]. The neotropical bat *Thyroptera tricolor* shows individuality in social calling behaviour, with individuals consistently differing in the number of contact calls they emit to maintain group cohesion [23].

Comprehensive studies on between-individual differences in bat behaviour, regarding not only vocalizations but personality differences in general, are scarce in the scientific literature. Only around a dozen studies have examined personality differences in bats, with a notable concentration on two temperate bat species, the little and big brown bat (*Myotis lucifugus* and *Eptesicus fuscus*) [24–28]. In all existing studies, bats exhibited repeatable behaviours in exploration [24], boldness [29], aggression [25] or social tendencies [27]. In tropical nectarivorous bats, wild *Glossophaga commissarisi* displayed consistent individual behaviour in a foraging context [30]. Recent studies have revealed that the closely related *Glossophaga soricina* is a species complex with at least four distinct species (*G. antillarum, G. valens, G. soricina* and *G. handleyi*), the latter comprising two lineages (*G. mutica* and *another lineage*) [31]. Wild *Glossophaga soricina handleyi* and captive *Glossophaga soricina antillarum* show notable behavioural variations in foraging strategies and territorial behaviour [32,33]. Certain individuals consistently and effectively monopolize and defend specific plants by aggressively chasing away intruders, accompanied by high-pitched chattering vocalizations. In contrast, other individuals engage in trap-lining foraging, thereby probing and feeding on various plants along a known route [32]. This strongly suggests the presence of significant individual differences in both classical behavioural traits and vocalization behaviour in *G. soricina*. Additionally, a thorough vocal repertoire for captive *G. s. antillarum* bats has been described and indicates a highly developed vocal communication system [34]. Given the prevalence of *G. soricina* as one of the most common bat species in Latin America and its amenability to extended housing [35], we capitalized on these factors to investigate the relationship between vocalizations and behavioural variation in *G. s. handleyi*.

In this study, our objective was to examine whether social calls are indicative of a certain behavioural type. To this end, we (i) aimed to demonstrate that consistent between-individual variation in bats comprises spatial (i.e. movement), as well as vocal behaviour. We then explored (ii) how individual vocalization levels relate to classic personality traits and whether there is a relationship between social calling behaviour and a certain behavioural type. Therefore, we developed a testing regime where we repeatedly exposed individual bats to three different experimental situations: a NE test, a novel object (NO) test and a foraging under risk (FUR) test. This setup allowed us to assess individual behavioural responses, both spatial and vocal, towards novel and risky stressors. Repeated measures across time and contexts enabled us to estimate the level of variation caused by between-individual differences, i.e. personality traits. We hypothesized that bat behaviour would be individually repeatable and conform to personality traits. Moreover, we hypothesized that individuals would exhibit consistent differences in the level of social call production. However, we did not have a specific *a priori* hypothesis regarding which behavioural type would be more inclined to produce social calls. Additionally, we conducted control experiments (iii) to validate that our measurements accurately reflect the intended traits, rather than representing artefacts from underlying behavioural processes. Conducting control experiments in animal personality studies to validate experimental approaches has been suggested [36] but is seldom employed in this research field. Here, we expected to find significant differences in averaged behaviours between test and control situations.

## 2. Methods

### (a) General fieldwork

We tested 60 wild male *G. s. handleyi* bats over the course of 2 years (April to June 2021 and January to May 2022) in the Santa Rosa National Park, Conservation Area Guanacaste (ACG), Costa Rica (10°50ʹ21.9″N 85°37ʹ05.2″W). Work was conducted under permits no. ACG-028-2021 and ACG-PI-006-2022 issued by the Costa Rican National System of Conservation Areas (SINAC) and the Ministry for Environment and Energy (MINAE). All fieldwork complied with the laws in Costa Rica and the guidelines of Eurobats and IUCN.

### (b) Animal capture and husbandry

Bats were captured in consecutive groups of 7–11 individuals each (2021—group 1: *n* = 7, group 2: *n* = 9, group 3: *n* = 9; 2022—group 4: *n* = 6, group 5: *n* = 8, group 6: *n* = 10, group 7: *n* = 11; total: *n* = 60). All bats in a given group underwent the experiments before we captured and tested the next group of bats. Following the STRANGE framework [37], we used different capture methods to reduce capture bias: we caught bats during nighttime with mist nets (3 m height × 12 m length, mesh size 19 mm; Ecotone, Gdynia, Poland) in the forest, close to known feeding sites, or during the day with hand nets in day roosts in a total of 7 different sites inside the Santa Rosa National Park in Sectors Santa Rosa (10°48′46.0″N 85°38′36.8″W), Horizontes (10°42′51.2″N 85°35′46.6″W) and Poco Sol (10°52′42.7″N 85°34′22.1″W). While we cannot rule out a certain level of relatedness among bats captured in the same roosts, we expect relatedness to be low owing to a strong male-biased dispersal in this species [38]. Additionally, the heritability of personality traits is often low [39], which is why we consider a potential low relatedness to be negligible. Following species identification, which was performed with a taxonomic field key of Costa Rican bats [40], we determined sex, reproductive state, forearm length (manual caliper diaMax, ± 0.1 mm, Wiha Werkzeuge GmbH, Schonach, Germany) and weight (digital scale ± 0.1 g, Foraco, Lotus NL B.V., The Hague, Netherlands). Bats were marked with wing punches using a biopsy punch (DCtattoo, 1.5 mm Ø) for individual identification. After capture and marking, bats were housed in two large outdoor flight cages (Eureka!: Hexagon Screen House; 3.6 × 4.2 × 2.3 m, Johnson Outdoors Inc., Racine, USA). Each flight cage was equipped with one or two custom-made day roosts mounted on the ceiling and two cylindrical bird water feeders with a protruding opening. Bats were offered a diet of daily prepared nectar substitute (NektarPlus, Nekton GmbH, Pforzheim, Germany, mixing ratio 1 : 5 in tap water, resulting in a 17% sugar concentration) and sugar solution (organic brown sugar, Zukra, San José, Costa Rica, mixing ratio 1 : 5 in tap water, resulting in a 17% sugar concentration), both accessible *ad libitum* to the bats. There were no indications of negative effects in the sampled individuals and after data acquisition was terminated (mean captivity period: 21±6 days), we released all bats at their respective site of capture before we proceeded with the next group of bats.

### (c) Experimental setup and protocol

Following capture and marking, each group of bats was given an acclimatization period of two full nights in the housing cage, after which experimental testing started. All experiments were conducted at night (6.45 pm to 3 am) and followed a standardized routine: we captured an individual bat from the housing cage with a hand net and transferred it to the experimental site (<300 m distance) in a cloth bag. The experimental site was located inside a building, where we used a flight cage (Coleman: Octagon 8 family tent; 3.96 × 3.96 × 2.08 m, The Coleman Company, Inc., Chicago, USA) as the experimental arena (electronic supplementary material, figure S1). After transfer to the experimental site, an individual’s weight was assessed and the bat was given 2 min undisturbed in the cloth bag to acclimatize after being caught and handled. After the 2 min acclimatization period, the bat was hand-fed with a 17% sugar solution (to mitigate the impact of immediate hunger levels on behavioural responses) before being released into the experimental arena, which marked the start of a trial. Each trial lasted for 30 min, after which the bat was caught and transferred back into the second housing cage, and the next individual was captured from the first housing cage.

We assessed bats’ behavioural responses in three different experimental situations: NE test, NO test and FUR test, plus their respective controls: control NO (cNO) test and control FUR (cFUR) test. We conducted all five behavioural assays (i.e. experiments) twice per group: after all bats of a respective group had performed the first trial of each experiment, we repeated the experiments in the same order (i.e. second trial), resulting in an inter-trial interval of 8±2 days.

### (d) Behavioural assays

#### (i) Novel environment (NE) test

The NE test is a standard open-field test and assesses an individual’s behavioural response to an unknown environment. Given that an unknown environment can offer the potential of a threat (i.e. a predator) being present, we expected this situation to induce anxiety and stress in the focal individual. Elevated spatial locomotion during a stressful situation can be interpreted as a form of active avoidance, i.e. proactive coping [41]. Therefore, we value the behaviour expressed in the NE test as indicative of an individual’s stress-coping mechanism [42]. A control test for the NE test was not feasible due to the nature of the test and logistical constraints in the field.

#### (ii) Novel object (NO) test

In this test, an echo-acoustically conspicuous object was positioned 50 cm below the centre of the flight cage ceiling (electronic supplementary material, figure S1E). This setup targets the trait Exploration–Avoidance by assessing an individual’s willingness to approach or inspect a NO. The NO was either a ball made from rubber filaments, i.e. ‘koosh ball’, or a hollow hemisphere (electronic supplementary material, figure S2A, B), and varied between the first and second trial. The order of NOs presented during the two trials was pseudo-randomized and differed between groups but was the same for each individual within a group.

This test was complemented by a control (cNO) to determine whether responses occurred specifically owing to novelty: in the cNO test the NO was replaced by a familiar object (either a spikey massage ball or an artificial, oval-shaped 3D-printed object, electronic supplementary material, figure S2C, D). The object was made familiar to the bats through its continuous presentation in the housing cages, starting from the day of capture. Thus, each group of bats was familiarized with one object and that same object was used in both trials of the cNO test, while different groups were familiarized with different objects.

#### (iii) Foraging under risk (FUR) test

In the FUR test, we hung a cylindrical bird feeder with a protruding opening from the ceiling of the flight cage, offering a food reward (17% nectar substitute: NektarPlus in a 1 : 5 ratio with tap water) alongside a risk-stimulus: a torch (2-in-1-UV-lamp, Letion, London, UK) mounted at the flight cage ceiling (electronic supplementary material, figure S1F) that shone a white light beam (500 lumen) on the protruding opening of the feeder. Here, we aimed to capture variation in an individual’s willingness to move and forage in the presence of a risk stimulus (light), indicative of their inclination to engage in a risky situation [43], thus targeting the personality trait Boldness–Shyness.

During the cFUR test the light source was turned off, allowing us to verify that behavioural responses occurred owing to the element of risk rather than the feeding setup itself. Subjects were exposed to an additional night of this control setup before the actual cFUR test to avoid measuring responses to a novel feeding situation.

The order of experiments was pseudo-randomized between groups, with an exception for the NE test, which was always conducted first in all groups and repeated the following night (with inter-trial interval of 24 h to minimize habituation effects on behavioural responses), and the cNO test, which was always conducted last to maximize habituation effects towards the familiar object. We tested all subjects of a group individually once per night (in randomized order) using the same experimental setup. Different experiments were conducted three nights in a row, followed by one night of resting.

### (e) Data capture and coding

To capture the behavioural responses of the subjects, we used two infrared-sensitive camcorders. One of the cameras (DCR-SR190, Sony, Japan) was positioned on a tripod in front of the cage, allowing for a wide overview of the experimental arena (electronic supplementary material, figure S1B). A second camera (DCR-SR55, Sony, Japan) was positioned inside and focused on the experimental set-up from below (electronic supplementary material, figure S1D), allowing us to confirm approaches and interactions with the experimental stimuli. Experiments were conducted in darkness, illuminated only by infrared (IR) light (electronic supplementary material, figure S1C, AEGIS UFLED IR lamp, Bosch, Ottobrunn, Germany). An ultrasonic microphone (electronic supplementary material, figure S1D, Avisoft USG 116H with CM16 condenser microphone, frequency range 2−200 kHz ± 3 dB) connected to a portable PC (electronic supplementary material, figure S1A, Lenovo S21e-20) running Avisoft-RECORDER software (R. Specht, Avisoft Bioacoustics, Glienicke, Germany; v. 4.2.3102 from 16th of March 2021) recorded social vocalizations.

All video recordings were coded using BORIS (Behavioral Observation Research Interactive Software [44]). For the analysis, we virtually divided the flight cage into 17 different ‘sectors’ along the walls and ceiling of the arena. In each experiment, we coded several behavioural variables related to spatial movements: e.g. the duration of the first flight after being released from the observer’s hand, the time an individual spent in flight, the number of times an individual rested or perched somewhere in the flight cage and the number of different sectors a bat visited during the experiment. Additionally, we coded behavioural variables related to interacting with the experimental stimuli: e.g. the number of times an individual inspected the unknown object, fed from or approached the feeder, and the latencies thereof (see Ethogram in electronic supplementary material, table S1). From those coded behavioural variables, we calculated our response variables (electronic supplementary material, Table S2). All videos were coded by T.S. (primary observer) or R.C. (assistant observer). We calculated the inter-rater-repeatability (IRR) to ensure consistent coding between observers. After training the assistant observer, we reached an IRR of at least 95% for each behaviour separately. All audio recordings were analysed using Avisoft SASLab Pro (R. Specht, Avisoft Bioacoustics, Glienicke, Germany). Prior to analysis, we down-sampled the sampling frequency from 500 to 250 kHz and employed a high-pass filter of 10 kHz to remove noise. Spectrograms were generated using a 1024-point fast Fourier transformation, a frame size of 100% and a Hamming window with 87.5% overlap, resulting in a bandwidth of 317 Hz and a frequency resolution of 244 Hz. Audio and video recordings were synchronized via spoken commands by the observer. We recorded individual social calls during the experiments, which all resembled a specific call type classified for captive *G. s. antillarum*. Bats in captivity uttered those ‘alert calls’ whenever individuals appeared to be alert and attentive towards a situation and related to vigilant, watchful behaviours [34]. We identified and quantified these recorded alert calls using Avisoft’s call detection and template-based spectrogram comparison. First, we visually inspected audio recordings and manually extracted spectrogram images of the clearest social calls to generate a template spectrogram repertoire. We then used Avisoft’s event-based option to detect sound events above a predefined amplitude threshold (threshold: 0.2 V, hold-time: 0.01 s). Next, we conducted spectrogram image cross-comparisons between the detected call and reference templates from the template repertoire. We let Avisoft automatically label detected calls, which were then verified visually by the primary observer. Finally, we extracted the number of alert calls produced by the focal individual for each observation.

### (f) Statistical analysis

Owing to technical difficulties and electricity failure at the field site during the experiments, the recording of a few experimental trials failed, resulting in varying sample sizes throughout the study. A detailed sample size table can be found in electronic supplementary material, table S3.

As a preliminary analysis, we fitted linear mixed effects models (LMM) to identify variables that significantly affected behavioural responses, using all valid test data (*N*_NE_ = 101, *N*_NO_ = 79, *N*_FUR_ = 87). Significant fixed effects from the LMM models for each response variable were added as fixed effects to the univariate mixed-effects models for repeatability estimation (see below) to achieve the most accurate repeatability estimates. The statistical protocol and results for this preliminary, supportive analysis are detailed in the Method and Result section of the electronic supplementary material.

Repeatability scores (*R*) evaluate individual variation within a population by calculating the ratio of between-individual variance (*V*_ind_) to the sum of *V*_ind_ and within-individual variance (*V*_e_). We used all data with two valid repeats per individual (electronic supplementary material, table S3) and the rptR package [45] to compute adjusted repeatability scores (*R*_adj_) using univariate linear mixed-effects models [46]. Adjusted *R* scores control for confounding effects by including significant variables as fixed effects. Thus, all models were fitted with a behavioural trait as the response variable, *bat identity* as a random effect, and the respective significant variables assessed in the preliminary analysis (LMM) as fixed effects, while always including the fixed effect ‘Trial’ to control for habituation effects. We computed confidence intervals and *p*-values for *R*_adj_ using parametric bootstrapping with 1000 simulations. We evaluated model adequacy through visual examination of residual histograms, Q–Q plots, and fitted versus residual plots. We applied a Benjamini–Hochberg procedure to correct for multiple testing. We also constructed LMM models with the lme4 [47]package to extract variance components (*V*_ind_ and *V*_e_) for each response variable. We then averaged repeatable behavioural variables per individual across both trials, resulting in one final score per individual and experiment. This approach minimizes the impact of varying external factors and extreme phenotypes [48]. Given our high repeatability scores (see §3), we believe that averaged trait scores accurately reflect the fraction of the variation attributable to individual differences, i.e. personality traits.

To assess the relatedness of variables within tests, we conducted three separate principal component analyses (PCAs) on the spatial behavioural variables from each experiment. We included only trials with actively participating bats (percentage flying > 1%), resulting in sample sizes of *N*_NE_ = 57, *N*_NO_ = 45 and *N*_FUR_ = 52. We confirmed sampling adequacy with the Kaiser–Meyer–Olkin Test (KMO ≥ 0.501) and Bartlett’s test of sphericity (*p* < 0.001) [49]. We entered 7, 8 and 11 variables into the PCAs for the NE, NO and FUR tests, respectively, and extracted two components each (electronic supplementary material, table S5), as suggested by the parallel analyses. The PCA solution was Varimax-rotated and variable loadings >±0.4 were considered salient (electronic supplementary material, table S7). All subsequent analyses used individual scores from the PCA loadings.

We constructed a correlation matrix with both principal components (PCs) from each of the three experiments using Pearson’s correlation coefficients to assess a potential behavioural syndrome structure. A frequent caveat of behavioural syndrome research is the necessity for partitioning within- and among-individual correlations because only the among-individual correlations constitute a behavioural syndrome, while within-individual correlations represent individual ‘correlational plasticity’ levels [50]. The state-of-the-art method is a Bayesian multivariate modelling approach, where both sources of covariation can be disentangled and analysed separately. However, Bayesian models are very data-hungry and thus inapplicable in many ecological field studies [51]. We here circumvent this caveat by using the retrieved PCs to calculate correlations. As the PCs are constructed from averaged trait values, there is no within-individual correlation present in the components. We can thus investigate the between-individual correlations (i.e. behavioural syndrome) by calculating Pearson’s correlation coefficient of the PCs. We limited our dataset to those observations where a focal individual was active during all experiments, reducing our sample size to *n* = 43.

In the main step of the analysis, we assessed whether spatial behaviours (represented as PCs) could predict the social calling behaviour of individual bats. For each experiment separately, we fitted models with individual calling scores (averaged across trials and rounded to integers) as response variables and individual PC scores as fixed effects. Given that our dataset comprised over-dispersed count data with a high frequency of zero values (indicating no social calls recorded), we employed hurdle regression as our modelling approach, using the ‘glmmTMB’ package [52]. Hurdle models comprise two components: a binomial probability model, termed the ‘zero component’, and a count data model, referred to as the ‘conditional component’, which is truncated at zero values [53]. The ‘zero component’ predicts the occurrence of calling, i.e. whether the ‘hurdle’ for social calling has been surpassed. Conversely, the ‘conditional component’ predicts the count of calls when calling occurs [54], i.e. positive values reflect variability in the number of calls produced by individuals that called. For the ’zero component’, we employed binomial models with logit links, whereas for the ‘conditional component’, we utilized negative binomial models with log links.

Finally, we compared how individuals scored in the test and control situations to evaluate our methodology. We used Wilcoxon signed rank tests to assess differences in the mean number of object inspections between the unknown (NO) and familiar objects (cNO). Additionally, we compared the mean number of feedings between the risky (FUR) and non-risky (cFUR) situations.

The datasets and code used in the current study are available through the Open Science Framework (OSF): https://osf.io/ewu9r/?view_only=34258b88596f49ba80810ef88187bf98 [55].

As the statistical procedure we present here is complex and resulted in lengthy tables, we have moved some details of the results to the electronic supplementary material. Table 1 summarizes the analysis conducted and indicates where the results can be found.

**Table 1.** Overview of the statistical analysis with details of where the results are presented. ESM,electronic supplementary material.

analysis	type of result	results shown in	table/figure
LMM	preliminary	ESM	electronic supplementary material, tables S4 and S5
repeatability	main	main text and details in ESM	electronic supplementary material, table S6
PCA	main	main text and details in ESM	electronic supplementary material, table S7
correlation between PCs	main	main text and details in ESM	figure 2; electronic supplementary material Figures S4 and S5
hurdle model*s*	main	main text	table 2, figure 3
Wilcoxon signed rank test	main	main text	figure 4

**Table 2.** Results from hurdle models used to assess the relationship between personality traits (captured as PCs) and social calling behaviour for each experiment separately. IRR = incidence rate ratios, CI = confidence intervals, *p* = *p*‐value. Significant values are in bold.

	NE—# social calls			NO—# social calls			FUR—# social calls		
*predictors*	*IRR*	*CI*	*p*	*IRR*	*CI*	*p*	*IRR*	*CI*	*p*
**conditional model**	***n* = 20**			***n* = 17**			***n* = 18**		
(intercept)	107.46	63.88–180.76	**<0.001**	38.04	7.07–204.54	**<0.001**	15.79	0.52–479.41	0.113
PC1	0.84	0.53–1.35	0.479	3.24	0.76–13.74	0.111	3.77	0.41–35.04	0.243
PC2	**1.59**	**0.96–2.62**	**0.071**	1.08	0.57–2.04	0.810	0.83	0.24–2.92	0.772
**zero-inflated model**	***n* = 57**			***n* = 45**			***n* = 52**		
(intercept)	1.45	0.82–2.54	0.200	1.18	0.63–2.21	0.609	2.54	1.26–5.09	**0.009**
PC1	**0.49**	**0.27–0.91**	**0.024**	**0.47**	**0.23–0.95**	**0.035**	**0.32**	**0.15–0.70**	**0.004**
PC2	0.81	0.46–1.40	0.446	0.78	0.41– 1.48	0.446	1.38	0.71–2.69	0.345

## 3. Results

**Figure 1.**
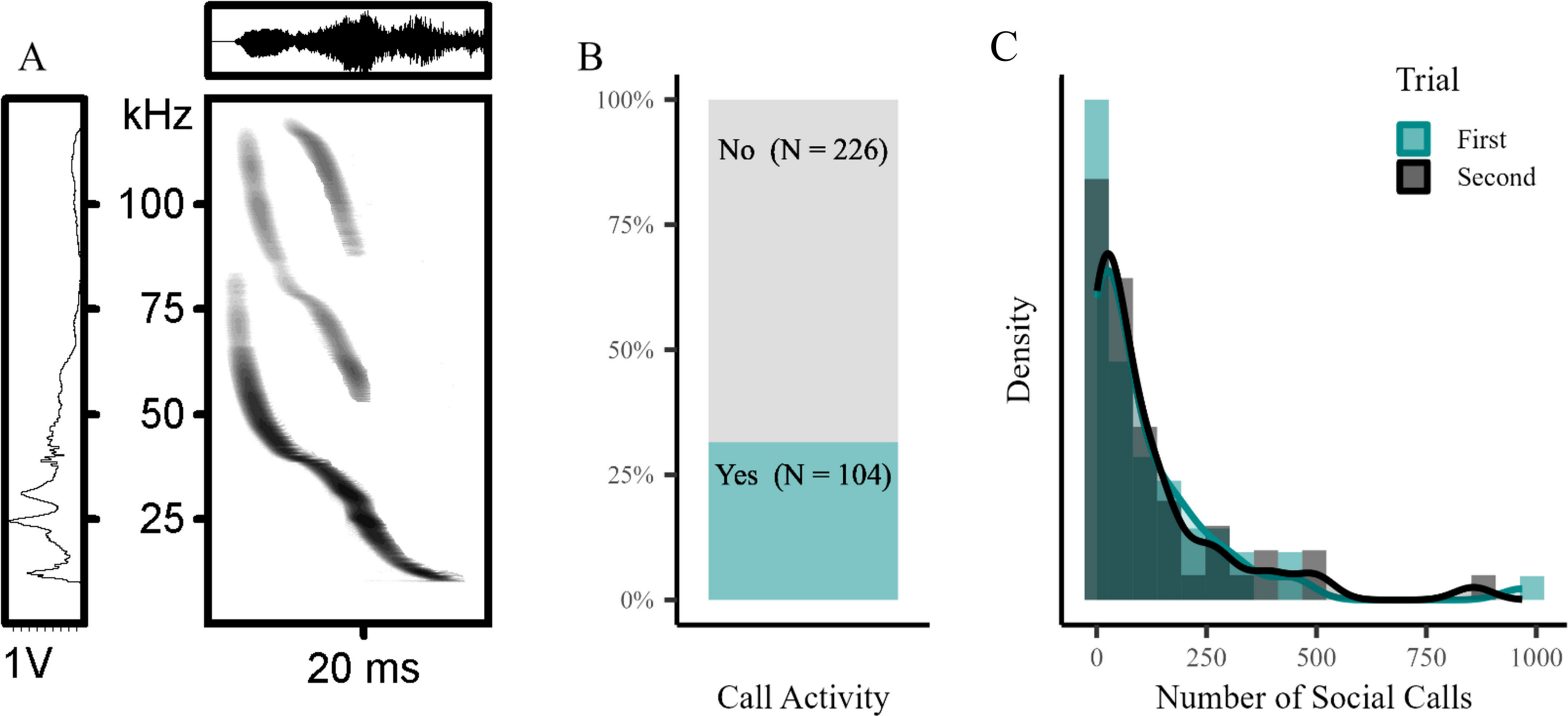
Alert calls and their prevalence among *G. s. handleyi*. (A) Oscillogram (upper panel) and spectrogram (central panel) images of an alert call, showing time on the *x*-axis and voltage and frequency on the *y*-axis, respectively. The left panel depicts the power spectrum of the call, with intensity on the *x*-axis and frequency on the *y*-axis. (B) Call activity among all bats in all trials (*N* = 330). (C) Density and histogram plot showing the distribution of the number of social calls among the vocally active bats. Turquoise bars and line show the number of calls during the first trial, while grey bars and black line depict the second trial.

Bats exhibited substantial between-individual variation in all analysed behavioural responses in the three experimental settings (see histogram and density plots for all spatial variables from each experiment in electronic supplementary material, figure S3). Importantly, individuals differed not only in spatial but also in vocalization behaviour. Some bats consistently produced distinct, stereotyped social calls, all belonging to the same type formerly described as ‘alert call’ (figure 1*a*, electronic supplementary material, figure S6) [34]. In 104 out of 330 total trials an individual emitted at least one such social call (figure 1*b*), with great variance in the number of calls produced by vocally active bats (figure 1*c*).

### (a) Behavioural repeatability

Out of the 39 variables measured in the three experiments, 35 showed significant repeatability across trials, with adjusted repeatability estimates ranging from 0.31 to 0.98 (electronic supplementary material, table S6). We also assessed cross-contextual consistency for individual spatial activity and social calling behaviour by calculating mean values across trials and calculating repeatability scores across experiments. Both spatial activity (*R* = 0.932, *p* < 0.001) and social call production (*R* = 0.653, *p* < 0.001) were highly repeatable between individuals and across experiments.

### (b) Behavioural correlations within tests

#### (i) Novel environment test

We found two PCs, which cumulatively explained 76% of the variance (electronic supplementary material, Table S7). The first axis (PC1_NE_) represented a spectrum from highly mobile individuals exploring the unknown arena to more sedentary ones and we thus interpreted it as a **Proactiveness–Reactiveness** axis (PC1_NE_—number of perches: 0.92; percentage explored: 0.83; duration of first perch: −0.57; duration of longest perch: −0.82; proportional variance: 43%). The second axis (PC2_NE_) represented variation in flight durations, ranging from highly restless individuals with extended flight times to more tranquil individuals with shorter flight durations. *G. soricina* is a highly active bat species [56] and expresses restlessness in elevated flight behaviour (M. Knörnschild , 2010, personal observation). Consequently, we interpreted this second component as an **Agitation–Tranquillity** axis (PC2_NE_—duration of first flight: 0.8; duration of longest flight: 0.92; proportional variance: 33%). The variable ‘percentage flying’ contributed significantly to both components (percentage flying PC1_NE_: 0.67; PC2_NE_: 0.68). This result becomes more intuitive when considering that the primary mode of locomotion in those bats is almost exclusively flight. Thus, both behavioural responses, i.e. how proactively/reactively an individual deals with novelty-induced stress and how agitated an individual is, were expressed as modifications of flight behaviour.

#### (ii) Novel object test

Two PCs cumulatively explained 68% of the variance (electronic supplementary material, Table S7). PC1_NO_ varies from actively exploring individuals—both spatially and towards the unknown object—to ‘non-explorative’ individuals, and thus we termed this axis **Exploration–Avoidance** (PC1_NO_—number of perches: 0.87; percentage explored: 0.83; percentage flying: 0.81; number of object inspections: 0.46; duration of first perch: −0.52; duration of longest perch: −0.86, proportional variance: 43%). PC2_NO_ indicated an **Agitation—Tranquillity** axis, as in the NE test (PC2_NO_—duration of first flight: 0.82, duration of longest flight: 0.87, proportional variance: 25%).

#### (iii) Foraging under risk test

Two PCs cumulatively explained 69% of the variance (electronic supplementary material, table S7). PC1_FUR_ represented a cline from risk-prone individuals with more feedings, quicker approaches and increased locomotion during the risky situation, to more risk-averse individuals avoiding the illuminated feeder. We thus considered it a **Boldness—Shyness** axis (number of perches: 0.85; percentage explored: 0.86; percentage flying: 0.81; number of feedings: 0.72; number of approaches: 0.8; latency to approach: −0.8; duration of first perch: −0.57, duration of longest perch: −0.84, proportional variance: 50%). PC2_FUR_ constituted an **Agitation—Tranquillity** axis (PC2_FUR_—duration of first flight: 0.82, duration of longest flight: 0.83, proportional variance: 19%).

### (c) Behavioural syndrome structure

**Figure 2.**
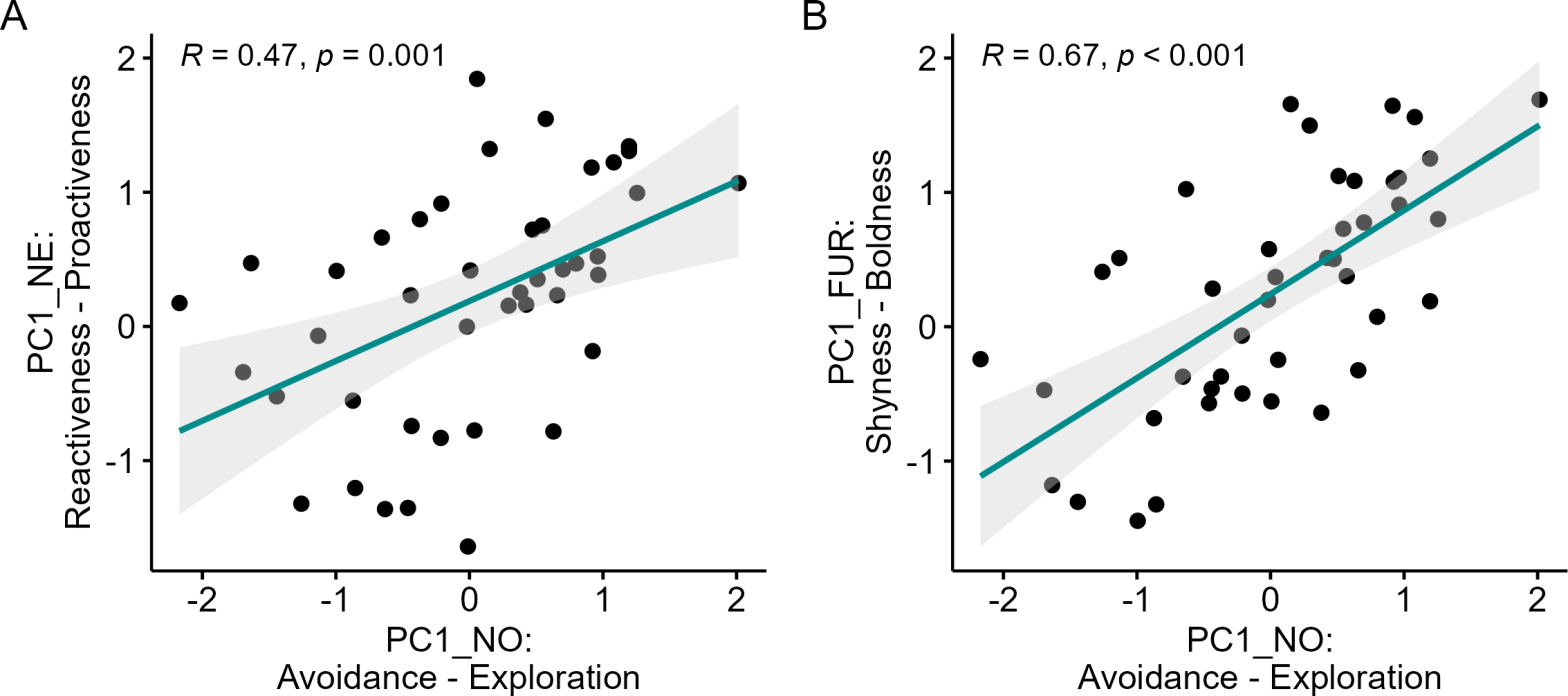
Scatterplots showing Pearson’s correlations between PC1s from different tests (*N* = 43). Dots depict single individuals, shaded areas around the trendlines show the 95% CI. (A) Strong positive correlation between Reactiveness–Proactiveness (NE test) and Avoidance–Exploration (NO test). (B) Strong positive correlation between Shyness–Boldness (FUR test) and Avoidance–Exploration (NO test).

We examined the relationship between PCs across different experiments to explore a potential behavioural syndrome structure in *G. s. handleyi* (electronic supplementary material, Fig S4). We found strong positive correlations between PC1_NO_ and PC1_NE_ (*R* = 0.47, *p* = 0.001, figure 2*a*) indicating an **Exploration–Proactiveness** syndrome. Additionally, a strong positive correlation between PC1_NO_ and PC1_FUR_ (*R* = 0.67, *p* < 0.001, figure 2*b*) indicated an **Exploration–Boldness** syndrome. Interestingly, PC1_NE_ and PC1_FUR_ were uncorrelated (*R* = 0.24, *p* = 0.11, electronic supplementary material, figure S4). We found strong positive correlations between all PC2 scores (see electronic supplementary material, figure S5), indicating consistent agitation levels between individuals across experiments.

### (d) Does personality predict individual calling rates?

**Figure 3.**
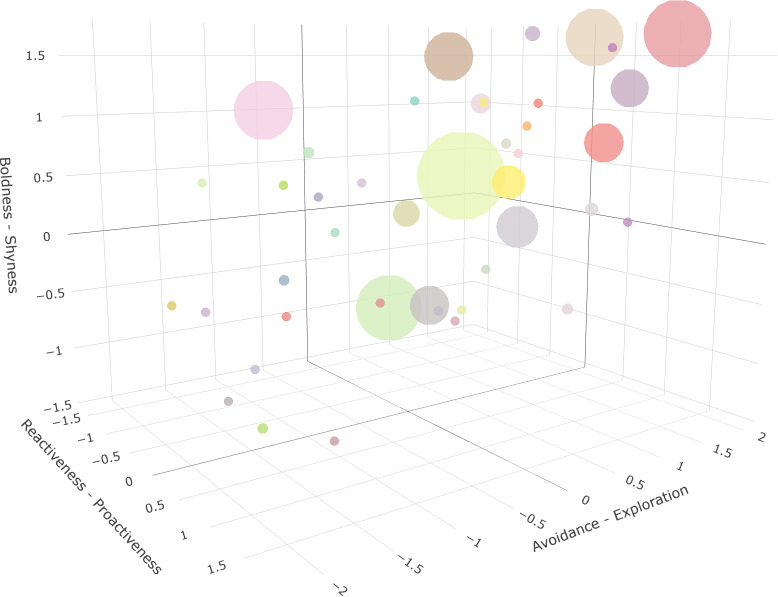
3D system of coordinates representing three major personality axes: Reactiveness–Proactiveness, Avoidance–Exploration and Shyness–Boldness derived from the first principal components of the NE, NO and FUR test, respectively. Negative values indicate higher levels of Reactiveness, Avoidance and Shyness, while positive values refer to higher levels of Proactiveness, Exploration and Boldness, respectively. Each bat is represented as an individually coloured dot, while dot size depicts the number of social calls produced (bigger dots indicate a higher numbers of social calls).

We constructed hurdle models to investigate whether personality traits (captured by PC scores) would predict individual social call production. Whether calling occurred was significantly influenced by PC1 scores of all three experiments (table 2, zero-inflated model). These results indicate that bats expressing higher levels of proactiveness, exploration and boldness were more likely to emit a social call during the experimental situations (figure 3). Additionally, and only in the NE test, we found a trend that more agitated bats produced more social calls in this experiment (table 2, conditional model).

### (e) Methodological validation

**Figure 4.**
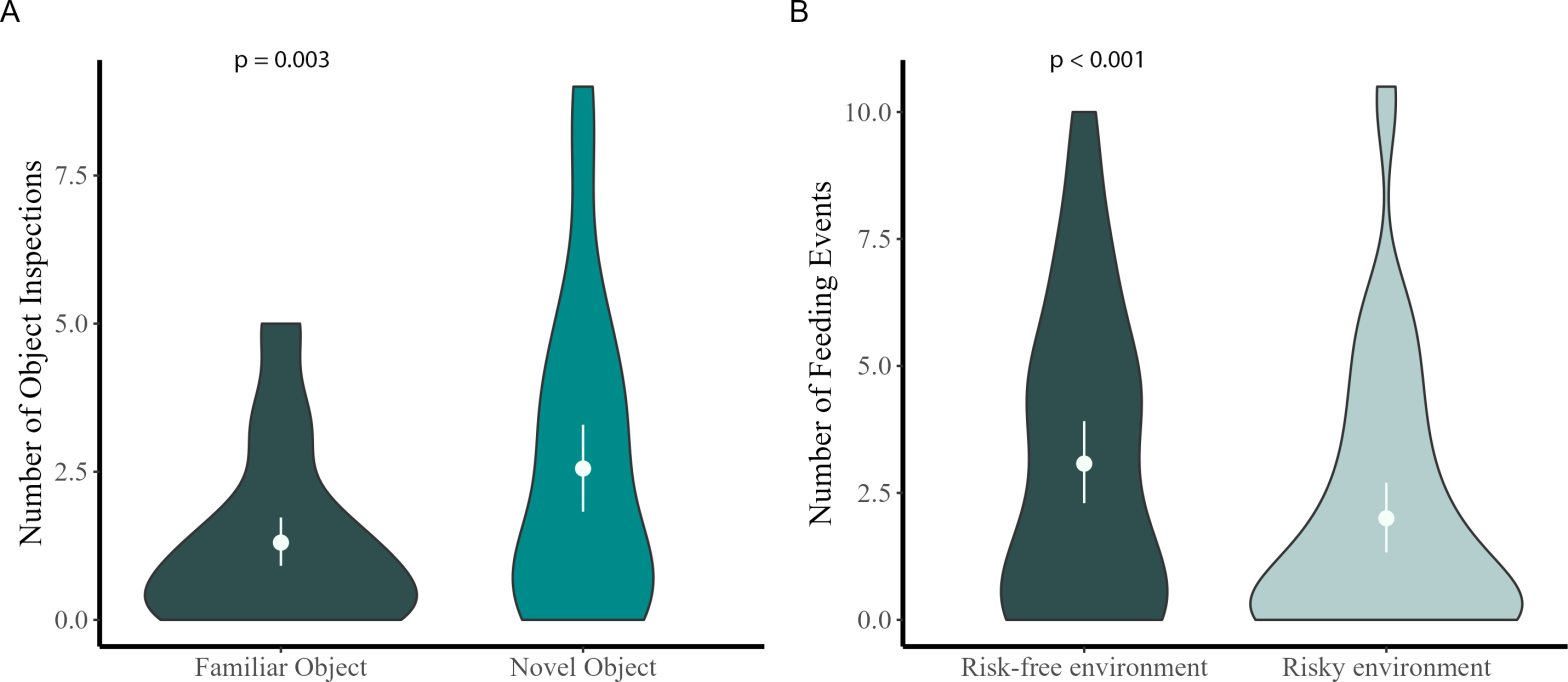
Violin plots depicting the mean number of (A) object inspections during cNO and NO (*N* = 46) and (B) feeding events during cFUR and FUR (*N* = 52). The numbers of object inspections and feeding events were averaged per individual across trials. The white, whiskered dots inside the violins depict the mean and 95% CIs. *p*-values of the Wilcoxon signed rank test are displayed in each panel for the respective test.

Standardized personality tests for bats are currently lacking, thus we aimed to demonstrate the validity of our experimental approach. As predicted, bats inspected the familiar object significantly less than the NO (*W* = 693.5, *p* = 0.003, *n* = 46, figure 4*a*). This suggests that the number of object inspections reflects true exploratory behaviour rather than, e.g. pure locomotion activity. Similarly, bats foraged significantly more in the non-risky setup compared with when the light was turned on (*W* = 111, *p* < 0.001, *n* = 52, figure 4*b*). This indicates that the chosen stimulus (light) indeed posed a threat to the bats and the responses measured in the FUR test truly reflect boldness tendencies. Overall, results from control tests support our interpretation of the traits measured and validate our experimental approach.

## 4. Discussion

Animals use vocalizations for a variety of contexts and purposes, most of which have crucial implications for sociality, reproduction and survival [1,2], especially so in many bat species owing to their nocturnality and high sociability [1,2,15]. However, that vocalization activity may be part of an animal’s behavioural type has not gained widespread attention yet. Here, by employing a newly developed testing regime for nectarivorous bats, we reveal that *G. s. handleyi* males consistently differ in their production of a certain social call (so-called ‘alert calls’). Interestingly, these calls were indicative of distinct and verified personality traits: more proactive, exploratory and bold individuals demonstrated a higher likelihood of producing a social call during the experiments. This partly contrasts results from social call production in rodents: in mice, a species review (genus *Peromyscus*) showed that bolder species produced fewer agonistic ultrasonic barks whereas less bold species barked more often, potentially as a strategy to decrease interactions with conspecifics and thus avoid close encounters [57]. However, female laboratory rats expressed strong between-individual differences in their production of ultrasound vocalizations reflecting positive affective states towards a reward-indicating cue [58]. The emission of ultrasonic vocalizations was more likely in rats with a lower disposition for anxiety, and rats with higher numbers of calls had higher tendencies to attribute incentive salience to reward cues [58], which was previously associated with dopamine signalling [59]. Modulations of the dopaminergic system are tightly intertwined with stress-related behaviours [60], indicating a potential relationship between vocalization and stress-coping mechanisms. In our study, increased vocal production could be similarly associated with a certain coping style, namely proactive coping.

Behaviours associated with a proactive coping style typically include active responses to stressors, such as aggression or exploration. These individuals tend to exhibit behaviours aimed at confronting or actively coping with challenges, rather than avoiding them [41]. Here, proactive individuals in the NE test (characterized by high levels of spatial activity) were more likely to produce social calls. Their calling behaviour could thus be part of an active stress response, where individuals actively try to emit stress from their system, using both vocalizations to express their arousal and high levels of spatial locomotion in potentially searching for an escape. Conversely, highly risk-sensitive, i.e. shy, individuals might be expected to have lower thresholds for emitting alert signals in response to a stress stimulus [61]. However, we found that individuals with high boldness scores were more likely to produce alert calls, indicating that the vocalization behaviour might indeed be an expression of a bold, i.e. risk-prone instead of risk-averse, phenotype. Some bats produced extremely high numbers of calls during the experimental situation (maximum number of calls: ~1000 calls in 30 min), thereby constantly broadcasting their location. This constant dissemination of location-based information can indeed be recognized as bold behaviour *per se*. Lastly, our findings align with reports from the neotropical bat species *T. tricolor*. In this species, individuals hold a consistent role (vocal vs non-vocal individuals) within their social groups [23]. Interestingly, Sagot *et al.* [62] demonstrated that an individual bat’s vocalization behaviour predicted its exploration efforts, with more vocal bats finding more roosting opportunities in their natural habitat. Similarly, male *G. s. handelyi* bats that displayed higher levels of neophilia in the NO test were more likely to produce an alert call. However, in our study system, the alert calls do not have a direct function in exploratory behaviour, as in *T. tricolor*, and more research is needed to fully comprehend the purpose of the social calls we recorded here.

Among the vocal bats, individuals that expressed higher levels of agitation produced more social calls during the NE test. Additionally, we noted a decline in social call production over extended periods of captivity, potentially owing to a decrease in agitation caused by novel experiences like exposure to experimental conditions. The notion that acoustic signals can convey information about the agitation or arousal of the caller has been suggested in the context of within-individual variation in other bat species [63,64]. Here, considering between-individual differences, we believe a similar mechanism could be at play, where the experience of increased agitation leads to increased calling behaviour in a certain behavioural type.

In our study, vocal production differences between individuals were indicative of a certain behavioural type, raising the possibility that eavesdropping conspecifics can assess each other’s personality from a distance, which could influence social interactions critically. Through eavesdropping, animals can acquire information about another individual’s social status and relationships without direct interaction [65]: for instance, baboons and other primates use overheard vocalizations from conspecifics to infer rank relationships and behavioural tendencies, which are likely to correlate with personality traits, such as aggression [66]. Thus, it is an intriguing thought that individual differences in vocal behaviour are recognized and potentially informative to the receiver, whether intended or not. Consequently, the social selection pressures on specific behavioural types might shift, with the potential to affect individual fitness by altering group dynamics, social hierarchies or direct encounters between conspecifics. However, these ideas remain speculative as we did not assess the effects on social interactions in the current experimental set-up. Thus, more research is warranted to understand the potential ecological and evolutionary implications of increased behavioural predictability of certain individuals via vocalizations. We hope that future studies take advantage of this easily observable and readily quantifiable measure and investigate how links between vocalization and consistent behavioural differences shape social encounters, for example through vocal networks [2,67]. Additionally, it remains open to explore whether these social calls are intentionally produced signals aimed at alerting or attracting conspecifics or unintentional cues stemming from arousal or agitation.

Furthermore, it would be interesting to investigate the metabolic costs of increased social calling; Ophir *et al.* [68] demonstrated that vocalizing animals may expend up to eight times more energy than silent ones. In bats, it is known that echolocation calls are extremely energetically costly, if not produced during flight [69–71]. Knowledge about metabolic costs of social calling, however, is still extremely limited. In *T. tricolor*, stationarily produced social calls increase the energetic expenditure of the vocalizing bat substantially [72]. Thus, investigating potential differences in metabolic rates between individuals of *G. s. handleyi* could facilitate understanding the proximate mechanisms driving vocalization behaviour.

## 5. Conclusion

Our study revealed that consistent between-individual differences extended beyond spatial responses to vocal production rates. More exploratory, bold and proactive bats were more likely to produce social calls in experimental settings, and among the vocal bats, more agitated individuals produced higher numbers of calls. These results suggest that vocalization behaviour can be indicative of certain behavioural type, which has the potential to alter social interactions, group dynamics and consequently, social selection pressures.

## Data Availability

The datasets and code used in the current study are available through the Open Science Framework (OSF) [73]. Supplementary material is available online [74].
